# Context Dependence Signature, Stimulus Properties and Stimulus Probability as Predictors of ERP Amplitude Variability

**DOI:** 10.3389/fnhum.2019.00039

**Published:** 2019-02-26

**Authors:** Carlos Mugruza-Vassallo, Douglas Potter

**Affiliations:** ^1^Grupo de Investigación de Computación y Neurociencia Cognitiva, Facultad de Ingeniería y Gestión, Universidad Nacional Tecnológica de Lima Sur – UNTELS, Lima, Perú; ^2^Neuroscience and Development Group, Arts and Science, University of Dundee, Dundee, United Kingdom

**Keywords:** attention, event related potential (ERP), goal-driven network (GDN), MisMatch Negativity (MMN), P3a, stimulus-driven network (SDN), schizophrenia, sound properties

## Abstract

Typically, in an oddball paradigm with two experimental conditions, the longer the time between novels the greater P3a amplitude. Here the research question is: Does an oddball paradigm maintain the greater P3a amplitude under several experimental conditions? An EEG study was carried out with an oddball number parity decision task having four conditions in control and schizophrenic participants. Contrary to previous findings (Gonsalvez and Polich, 2002; Polich, 2007) in control participants, non-correlation was found between the time of a novel (N) stimulus condition to the next novel condition and P3a amplitude. Moreover, with an innovative method for stimulus properties extraction features and EEG analysis, single trial across-subject averaging of participants’ data revealed significant correlations (*r* > 0.3) of stimulus properties (such as probability, frequency, amplitude, and duration) on P300, and even *r* > 0.5 was found when N was an environmental sound in schizophrenic patients. Therefore, stimulus properties are strong markers of some of the features in the P3a wave. Finally, a context analysis of ERP waves across electrodes revealed a consistent modulation in novel appearance for MisMatch Negativity in schizophrenia. A supplementary analysis running linear modeling (LIMO) in EEG was also provided (see Supplementary Material). Therefore, in a multiple condition task: stimulus properties and their temporal properties are strong markers of some of the features in the P300 wave. An interpretation was done based on differences between controls and schizophrenics relate to differences in the operation of implicit memory for stimulus properties and stronger correlations were observed within groups related contextual and episodic processes.

## Introduction

The current view is that cognitive impairment in medicated schizophrenic patients is partially the result of impairments of attention control (Laurens et al., 2005) in the form of reduced efficiency of goal-driven control mechanisms (GDN) and a possible enhancement of sensitivity of stimulus-driven control mechanisms (SDN) to distractor stimuli (Corbetta and Shulman, 2002). To test this, an oddball task based on cues and targets was tested to test SDN and GDN.

The finding that a cue stimulus preceding a goal stimulus by a fixed interval speeds up response time is one of the oldest phenomena reported in psychology (e.g., Wundt, 1880 cited in Hackley, 2009). The effect also works across modalities (Bertelson, 1967; Bertelson and Tisseyre, 1968; Davis and Green, 1969). Studies have shown this effect in blocked designs, where a cue always announces the upcoming presentation of a target and precedes it by a fixed amount of time (e.g., Woodrow, 1914; Näätänen, 1970). This type of non-spatial cue warns the participant of the upcoming target. Whether the cue results in the alerting or alerting and orienting of attention to a particular point in time is not clear (Posner and Rothbart, 2007; Hackley, 2009). Moreover, in auditory-visual cross-modal tasks, changes in reaction times (RT) were interpreted as due to a auditory distraction in attention tasks (Corral and Escera, 2008). As Parmentier and colleagues have pointed out, one needs to note that orienting paradigms were not done in mixed blocks where targets do not always follow warnings or only do so after a temporal interval varying from trial to trial (Parmentier et al., 2010). Parmentier and colleagues hypothesized that an orienting response to a novel stimulus may be influenced by the informational content of the sound in a particular context. They explored this hypothesis in a three experiment between-subject study: (a) In the first ‘Informative’ experiment, standard (*p* = 0.8) and deviant (*p* = 0.2) tones always predicted a visual digit 250 ms later. (b) In the second ‘Uninformative’ experiment, the tones predicted a visual digit at 150, 250, or 350 ms only 50% of the time. (c) In the ‘Informative Deviant’ experiment, the (*p* = 0.8) standard tones predicted a visual digit 50% of the time and the (*p* = 0.2) deviants predicted a visual digit 100% of the time. In each case the digit had to be categorized as odd or even. They found in the ‘Informative’ condition that when the deviant stimulus predicted targets at the same rate as standard stimuli then RTs were slower to deviants. In the second ‘Uninformative’ experiment, in which standards and deviants did not differentially predict the timing of visual digits, they found no difference between the RTs. In the final experiment, in which standard stimuli only predicted visual digits 50% of the time but novel stimuli predicted visual digits 100% of the time, they found that deviants now improved RTs. Therefore, the results suggested that distraction is not present for deviant sounds with low information content, and that deviant sounds can improve the performance when these deviants carry additional information not contained in the standard stimuli (Parmentier et al., 2010).

Novel events are believed to be responsible for a pattern of responses marked by specific brain event related potential (ERP) waves typically obtained by ERP substraction: first, the automatic novelty-detection response or MisMatch Negativity (MMN; e.g., Näätänen et al., 1978; Näätänen and Winkler, 1999; Picton et al., 2000); second, the involuntary orientation response (P300; e.g., Grillon et al., 1991; Näätänen and Teder, 1991; Woods, 1992; Friedman et al., 2001). These unexpected novel sounds produced measurable behavioral effects such as longer RTs and a distinctive pattern of ERP deflections that include the MMN (e.g., Schröger, 1996), and the P3a (e.g., Woodward et al., 1991) as suggested by Näätänen (1991).

The oddball task is one of the most reported paradigms in the literature. In the oddball task, when the Inter-Stimulus Interval (ISI) is constant, the longer the non-target sequence length, the greater the P300 amplitude will be to a target stimulus (Gonsalvez et al., 1999). Moreover, in an extensive review of P300 research, Polich (2007) stated that a novel or deviant distractor produces a larger P300 called a P3a response. These P300 changes are interpreted as possible markers of attention activation and subsequent alterations of the content of short-term and long-term memory (Polich, 2007).

There is a strong P3a response at a low novel probability of 25% (classical Posner probability) or at lower probability, such as 15% (e.g., Potter et al., 2001) and the magnitude of the response is influenced by the task relevance of novel stimuli even at local probabilities of 50% (Parmentier et al., 2010).

Early studies of visual and auditory P300 have suggested that the auditory P300 is more sensitive to schizophrenia than the visual P300 (Ford, 1999; Jeon and Polich, 2003), and that the goal-driven attention processes reflected by target P3b may be particularly sensitive to higher-order cognitive deficits in schizophrenia relative to the stimulus-driven processes that may contribute to the P3a signal. P300 (P3b) has been proposed as a biological marker in schizophrenic patients because the P3b amplitude was reduced (McCarley et al., 1991). The model of P300 wave generators suggested by Polich proposed the activation of anterior cingulate structures for P3a and activation of temporo-parietal structures for P3b (Polich, 2007). Mathalon and colleagues aimed to have a more complete framework in their study of the sensitivity of the P3b and P3a in auditory and visual oddball paradigms to the effects of schizophrenia. A direct comparison of visual and auditory P3a and P3b failed to support the suggestion of differential sensitivity in schizophrenia. Their results suggest that the P300 is reduced and delayed in schizophrenia to the same degree in both sensory modalities and that the same attention system is engaged (Mathalon et al., 2010).

In an attempt to draw a more direct comparison between ERP markers and cognition, Kirihara and colleagues compared healthy subjects (*n* = 58) and schizophrenic patients (*n* = 60) in a three-tone oddball task (40 target stimuli and 200 standard stimuli and 40 novel stimuli) and calculated correlations between P300 amplitude (P3a at Cz; P3b at Pz) and scores in the Comprehension Index of Positive Thought Disorder (CIPTD). They found significant correlation of P3b (*r* = -0.322, *p* = 0.012) and non-significant correlation of P3a (*r* = 0.088, *p* = 0.609) with a mean peak P3a at Fz of 11.15 μV ± 4.4 μV in controls and 8.75 μV ± 5.7 μV in schizophrenics. Both correlation results are supported by the idea that the frontal lobe activity generates P3a for attention processing while P3b is strongly linked to memory by the measure of CIPTD (Kirihara et al., 2009). Recent work has proposed to systematically study ERP markers after each therapy and use predictive coding in schizophrenia response (Mugruza-Vassallo, 2016). They also allow the visualization of differences in MMN responses around 100 ms between both groups.

Several studies explored the possibility of different activations in MMN in control and patients with cognitive impairment. For example, for deviant tones in an auditory task, the MMN was more prominent at frontal and right temporo-parietal electrodes in control participants and more frontal or frontal and central in medicated and non-medicated Parkinson disease patients, respectively (Solís-Vivanco et al., 2011). In schizophrenic patients, Näätänen and Kähkönen reviewed several MMN articles and found that MMN attenuation is in the temporal lobe for positive disease and in the frontal lobe for switching attention (see review by Näätänen and Kähkönen, 2009).

Many of the paradigms (e.g., Terrasa et al., 2018) manage probability using two or three conditions rather than the two conditions in the Posner’s original experiments (Posner et al., 1980). The paradigm that we decided to explore here has four conditions, therefore not only can we carry out more analysis between conditions but we can also study more effects of local probability in the switching of attention. The aim of the reanalysis of this data was to explore the effect of local probability on single trial P3a variance when a novel stimulus replaces the standard tone in the warning signal S1 and its link with MMN in the different conditions when the distractor is presented at different times at low local probability. Subsequently, the main analysis was to employ single trial analysis methods to determine whether the originally observed P3 effects can be enhanced by controlling for the effects of variables such as local probability as well as differences in the amplitude, duration or frequency content of the sound stimuli used in the task.

On the basis of the literature reviewed here it was hypothesized (H):

H1: Based on previous results regarding P3a amplitude in controls (Gonsalvez et al., 1999; Gonsalvez and Polich, 2002) and in schizophrenic patients (Kirihara et al., 2009), there will be a decrease in amplitude of P3a over time to novel stimuli (that replace the tone cue) as task duration (familiarity) increases, and that this will be greater in the control than in the schizophrenic participants.

H2: Based on previous results regarding P3a amplitude (Gonsalvez et al., 1999, 2002) and changes in RT due to informational content (Parmentier et al., 2010), the amplitude of P3a to novel stimuli (that replace the tone cue) will be systematically related to the local probability of novel stimuli as well as, to a lesser degree, fluctuations of frequency, amplitude and duration stimuli of immediately preceding cue, goal or novel stimuli.

H3: Based on previous findings on schizophrenic patients with regard to P300 amplitude (McCarley et al., 1991; Kirihara et al., 2009; Mathalon et al., 2010) and MMN modulation (Näätänen and Kähkönen, 2009), there will be a significant negative correlation between P3a amplitude on the current trial and the MMN on the subsequent trial. The rationale being that when the P3a to a novel stimulus is smaller, suggesting impaired context updating, then the ERP in the next trial shall be prone to produce a larger MMN to the next standard stimulus.

## Materials and Methods

### Participants

Thirty-four adults participated in this study: 21 healthy subjects (mean age: 36.1 ± 11.3 years; range 22–63 years) and thirteen schizophrenic individuals (mean age: 41.1 ± 11.1 years; range 22–60 years). All subjects were free from any history of auditory deficits or other known neurological illness. One healthy participant and one schizophrenic participant were excluded because there were too few usable segments of EEG data as a result of recording artifacts (<100 segments), leaving 20 healthy (20 right handed) subjects and 12 schizophrenic (12 right handed) subjects. All voluntary participants signed informed consent to participate in the study and were paid for their participation. All procedures performed in studies involving human participants were approved by the Research Ethics Committee of the University of Dundee in accordance with the ethical standards of the University of Dundee, that accord with the 1964 Helsinki declaration.

### Experiment Design

Subjects were asked to perform an odd/even number decision while their scalp EEG was recorded. The paradigm was composed of 600 trials, with trials chosen pseudo-randomly from one of four different conditions. Each trial consisted of a pair of sound stimuli. The parameters of the stimuli are given in Table 1. Participants were asked to respond by pressing a button as quickly as possible without sacrificing accuracy. One button was pressed when the number was odd, and another button was pressed when the number was even. Hand preference of response was counter-balanced across subjects. The Inter-Trial Interval (ITI) was 2300 ms. The task was presented in 5 separate blocks (120 trials each) with each of the four conditions presented in random order. Stimulus sequence was the same across all participants.

**Table 1:** Stimuli combinations for the experiment 1.

			Stimuli				
			
			SI		SOA	S2	
					
Stimuli name	Number of presentations	Code Processed	Type	*Time* (ms)		*Type*	*Time* (ms)
Standard goal stimuli	450	TG	Tone	50	300	Number	300
Novel only	50	TN	Tone	50	300	Novel	200
Simultaneous novel and goal	50	TNG	Tone	50	300	Number + Novel	300
Novel Preceding the goal	50	NG	Preceding novel	100	300	Number	300


*SOA, stimulus-onset asynchrony.*

### Stimuli

The sound stimuli were presented using Beyer Dynamic Headphones (DT 770) at 75 dB sound pressure level. Sounds files were stereo with 16-bit resolution and 22050 Hz sampling rate.

For the standard goal stimuli condition (TG), the first stimuli of each pair (S1) were 50 ms duration pure tones with 10 ms rise/fall times followed by a number sound (S2) of 300 ms duration.

For the novel only condition (TN), S1 were pure tones as in the TG condition, followed by a novel sound. These sounds were 100 ms in duration.

For the simultaneous novel and goal condition (TNG), S1 were pure tones as in the TG condition, followed by a number sound of 300 ms duration and a simultaneous laterally presented novel sound of 100 ms duration. These sounds were 300 ms in duration.

An innovative method for extracting sound properties and analysis was proposed and implemented. For the novel preceding the goal condition (NG), the first stimulus of each pair (S1) was either white noise (26 stimuli, 100 ms duration) or samples of environmental sounds (24 stimuli, 100 ms duration). An in-house Matlab script (detailed results of these calculations are not presented here) was used to calculate the following sound properties (see below). A correlation matrix was next computed to assess how the properties of the sounds related to each other (5,000 bootstrapped correlations with False discovery rate correction of *p*-values *p* = 0.05). From these results, an exploratory analysis to determine which of these sound properties modulated the P300 was conducted.

Extending duration, and intensity of one signal (Näätänen et al., 2012), 14 parameters were obtained from each pair of sounds S1 and S2:

R(n,1), R(n,2), and R(n,3): Fundamental frequency of S1, S2, and S1-S2 [i.e., R(n,3) = R(n,2) - R(n,1)].

R(n,4), R(n,5), and R(n,6): Sound durations of S1, S2, and S1-S2 [i.e., R(n,6) = R(n,5) - R(n,4)].

R(n,7): Average difference in the long term average spectrum (LTAS) between S1 and S2.

R(n,8): Normalized mutual information in frequency between S1 and S2.

R(n,9), R(n,10), and R(n,11): Mean amplitude in time of S1, S2, and S1-S2 [i.e., R(n,11) = R(n,10) - R(n,9)].

R(n,12), R(n,13), and R(n,14): Root mean square (RMS) in time of S1, S2, and S1-S2 [i.e., R(n,14) = R(n,13) – R(n,12)].

There are 14 parameters, 4 are exclusively for S1 which in the following results was the current cue or preceding novel and was compared with the other five sounds (current goal, previous goal, previous tone/preceding novel, previous novel on one of S1 or S2 and previous preceding novel) leaving the other 10 parameters per comparison in the left and right side, as seen in Table 2.

**Table 2:** Sound properties on the events of the experiment between control participants and schizophrenic patients.

Stimuli name	Stimulus property used for the calculi	Property seek in	
Freq(S1,R)	Frequency of S1	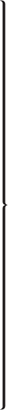	Current event
Dura(S1,R)	Duration of S1		
Rms(S1,R)	Root mean square (RMS) in time of S1		
Std(S1,R)	Standard deviation of S1		
Freq(S2,R)	Frequency of S2		
Freq(S1,R-S2,R)	Frequency of S1 – frequency of S2		
Dura(S2,R)	Duration of S2		
Dura(S1,R-S2,R)	Duration of S1 – duration of S2		
Ltas(S1,R,S2,R)	Average difference in the long term average spectrum between S1 and S2		
Entr(S1,R,S2,R)	Normalized mutual information in frequency between S1 and S2		
Rms(S2,R)	Root mean square (RMS) in time of S2		
Std(S2,R)	Standard deviation of S2		
Rms (S1,R-S2,R)	Root mean square in time of SI -Root mean square in time of S2		
Std(S1,R-S2,R)	Standard deviation of S1 – standard deviation of S2		
Freq(S2(t-l))	Frequency of the previous S2	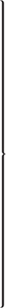	Previous event (previous S2)
Freq(S1,R-S2(t-1))	Frequency of S1 – frequency of the previous S2		
Dura(S2(t-l))	Duration of the previous S2		
Dura(S1,R-S2(t-l))	Duration of S1 – duration of the previous S2		
Ltas(S1,R,S2(t-l))	Average difference in the long term average spectrum between S1 and S2		
Entr(S1,R,S2(t-l))	Normalized mutual information in frequency between S1 and the previous S2		
Rms(S2(t-1))	Root mean square of the previous S2		
Std(S2(t-l))	Standard deviation of the previous S2		
Rms(S1,R-S2(t-l))	Root mean square in time of S1 -Root mean square in time of the previous S2		
Std(S1,R-S2(t-l))	Standard deviation of S1 – standard deviation of the previous S2		
Freq(S1(t-1))	Frequency of the previous SI	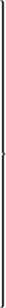	Previous event (previous S1)
Freq(S1,R-S1(t-1))	Frequency of S1 – frequency of the previous S1		
Dura(Sl(t-l))	Duration of the previous SI		
Dura(S1,R-Sl(t-l))	Duration of S1 – duration of the previous S1		
Ltas(S1,R,Sl(t-1))	Average difference in the long term average spectrum between S1 and the previous S1		
Entr(S1,R,Sl(t-l))	Normalized mutual information in frequency between S1 and the previous SI		
Rms(Sl(t-l))	Root mean square of the previous S1		
Std(S1(t-1))	Standard deviation of the previous S1		
Rms(S1,R-Sl(t-l))	Root mean square in time of S1-Root mean square in time of the previous S1		
Std(S1,R-Sl(t-l))	Standard deviation of S1 – standard deviation of the previous S1		
Freq(Nov(t-l)R)	Frequency of the previous novel, either on S1 or on S2	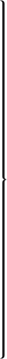	Previous novel, either on S1 or on S2
Fieq(S1,R-Nov(t-l)R	Frequency of S1 – frequency of the previous novel, either on S1 or on S2		
Dura(Nov(t-l)R)	Duration of the previous novel, either on S1 or on S2		
Dura(S1,R-Nov(t-l)R	Duration of S1 – duration of the previous novel, either on S1 or on S2		
Ltas(S1,R,Nov(t-l)R)	Average difference in the long term average spectrum between SI and the previous novel, either on S1 or on S2		
Entr(S1,R,Nov(t-1)R)	Normalized mutual information in frequency between S1 and the previous novel either om SI or on S2		
Rms(Nov(t-l)R)	Root mean square of the previous novel, either on S1 or on S2		
Std(Nov(t-l)R)	Standard deviation of the previous novel, either on S1 or on S2		
Rms(S1,R-Nov(t-1)R)	Root mean square in time of S1-Root mean square in time of the previous novel, either on S1 or on S2		
Std(S1,R-Nov(t-l)R)	Standard deviation of S1 – standard deviation of the previous novel, either on S1 or on S2		
Freq(Sl(PN)R)	Frequency of the previous novel on S1	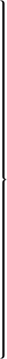	Previous novel on S1
Freq(S1,R-S1(PN)R)	Frequency of S1 – frequency of the previous novel on S1		
Dura(Sl(PN)R)	Duration of the previous novel on S1		
Dura(S1,R-Sl(PN)R)	Duration of S1 – duration of the previous novel on S1		
Ltas(S1,R,Sl(PN)R))	Average difference in the long term average spectrum between S1 and the previous novel on SI		
Entr(S1,R,Sl(PN)R)	Normalized mutual information in frequency between S1 and the previous novel on S1		
Rms(Sl(PN)R)	Root mean square of the previous novel on S1		
Std(Sl(PN)R)	Standard deviation of the previous novel on S1		
Rms(S1,R-Sl(PN)R)	Root mean square in time of Sl-Root mean square in time of the previous novel on S1		
Std(S1,R-Sl(PN)R)	Standard deviation of S1 - standard deviation of the previous novel on S1		


*Sound properties for right ear are shown (R: Right Ear), codes are similar for the left ear, changing R per L (L: Left Ear).*

### EEG Recording

Participants were seated in an armchair in a light and sound-attenuated room, and the keyboard was positioned near to their hands. EEG data were recorded with a BioSemiActiveTwo 32-channel EEG (BioSemi Inc., Amsterdam, Netherlands) acquisition system working with BioSemiActiView software (CortechSolutions). Amplified signals were digitized at 2500 Hz with 16-bit resolution. All electrode impedances were < 20 kΩ, the median resistance was 5 kΩ with only a few electrodes having higher resistance than 10 kΩ. The Active electrode system is more tolerant of higher impedance recordings and all channels were checked to ensure that noise levels were not excessive. Data were band-pass filtered between 0.2–500 Hz during data acquisition. Eye movements and blinks were recorded with two horizontal electrodes in the outer canthus of both eyes (HEOG) and two vertical electrodes in the infraorbital and supraorbital regions of the left eye (VEOG).

### Data Analysis

Goal conditions in this study are the standard goal stimuli (TG), the simultaneous novel and goal (TNG), and the novel preceding the goal (NG).

The RTs were analyzed using a 2 × 3 analysis of variance (ANOVA) using SPSS19 with groups as the between-subject factor and with goal conditions as the within-group factors.

**FIGURE 1:**
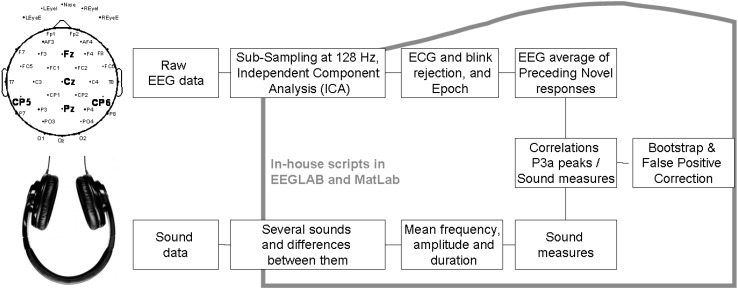
Block diagram of data processing in the first study.

EEG was analyzed following Figure 1. EEG pre-processing was conducted first through Polyrex (Polygraphic Recording Data Exchange, PolyRex, Kayser, 2003). Analyzer software (Brain Vision, LLC) was then used to down-sample the EEG data from 2500 to 128 Hz. After EEG-data were referenced to the mastoid, they were analyzed using EEGLAB (Delorme and Makeig, 2004) and Matlab in-house scripts. Eye-movements and artifacts were removed through independent components analysis (ICA, Makeig et al., 1997). Data were then filtered with a high-pass at 0.75 Hz and epoched from 300 ms before stimulus onset to 600 ms after stimulus onset. A baseline correction was then applied. The epochs were then checked for trials with excessive peak-to-peak deflections, amplifier clipping, or other artifacts.

The innovative EEG analysis considered three approaches that were taken to the analysis of the EEG data. In the first approach, to investigate the relationship between sound properties and the P300, single trial across-subjects averages were next computed for the 20 healthy participants and the peak amplitude between 350 and 450 ms of the Novel-Goal condition was taken as a measure of the P3a orienting response to the novel stimulus preceding the number decision. Correlations were next computed between amplitudes and the sounds properties (600 bootstrap percentile correlations) and a FDR correction for multiple testing applied (*p* < 0.05).

P3a amplitude measures from the EEG average in 20 controls and either sound properties or probabilities were then correlated using a bootstrap method (600 iterations) and a further correction of false positive of *p* < 0.05.

The purpose of the second analysis was to explore sources of variability of P3a deflection associated with the context of the immediately preceding trial. Seven conditions were identified and the ERP deflections to the second trial were computed for each subject. These were: standard goal followed by the standard (TG.TG), standard goal followed by the novel only (TG.TN), standard goal followed by the preceding novel (TG.NG), standard goal followed by the simultaneous novel and goal (TG.TNG), simultaneous novel and goal followed by the standard goal (TNG.TG), novel target followed by the standard goal (TN.TG) and preceding novel followed by the standard goal (NG.TG).

The ERP generated by the TG.TG condition was then subtracted from each of the other conditions to separate out the effects of the novel stimuli from the basic response to the number decision task. Therefore, within groups *t*-tests between each condition and the standard was run (*p* < 0.001) for significant differences at each time and for each channel.

## Results

### Behavioral Results

**FIGURE 2:**
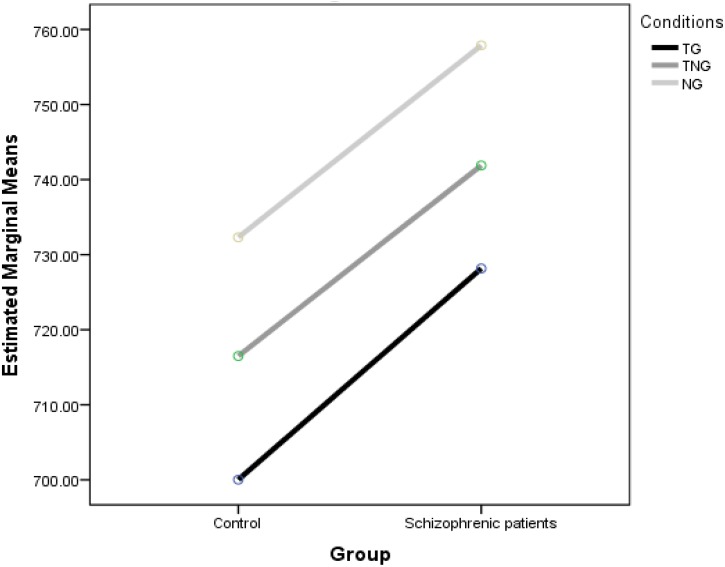
Effect of preceding (NG) and simultaneous (TNG) distractors on number parity decisions compared to simple number decision task (TG).

Reaction times for the standard goal stimuli (TG), novel preceding the goal (NG), novel target (TN) and simultaneous novel and goal (TNG) were analyzed. Figure 2 shows the mean RTs in each condition in control and schizophrenic patients.

Overall, participants performed well (94% accuracy of goal trials). The Group ANOVA of RTs yielded significant main effects of group [*F*(1,30) = 19.68, *p* < 0.001], schizophrenic patients showed delayed RTs. The Conditions ANOVA of RTs yielded significant main effects [*F*(2,60) = 13.28, *p* < 0.001]. This was due to differences between NG and either TG (difference of 30.96 ms at *p* < 0.001) or TNG (difference of 27.94 ms at *p* = 0.001) found in a *post hoc* test using Fisher’s least significant difference (LSD). In addition, there were no differences between TG and the other two goal conditions. Although significant differences were found, there was no significant interaction between Group and Condition [*F*(2,60) = 0.039, *p* = 0.962].

Overall, the small effect size in the differences in RT in the 2 × 3 ANOVA may be explained by the individual differences in pattern of the running average RTs in the different conditions (see Supplementary Material). Some individuals clearly showed distraction effects while others did not.

### EEG Results

**FIGURE 3:**
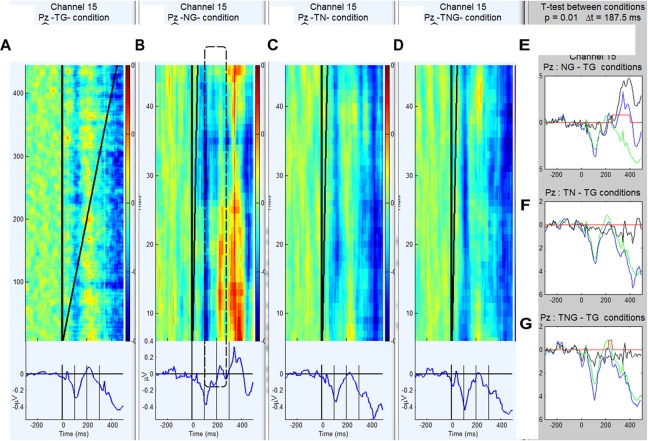
**(A–D)** Grand average ERP waveforms and trial by trial voltage plots at Pz electrode in 20 control participants in the standard goal (TG), novel preceding goal (NG), novel target (TN) and simultaneous novel and goal (TNG) conditions. **(E–G)** Waveforms generated by subtraction (in black) of novel conditions from control condition (TG in green) and corresponding *t*-values for successive time bins of 187.5 ms.

**FIGURE 4:**
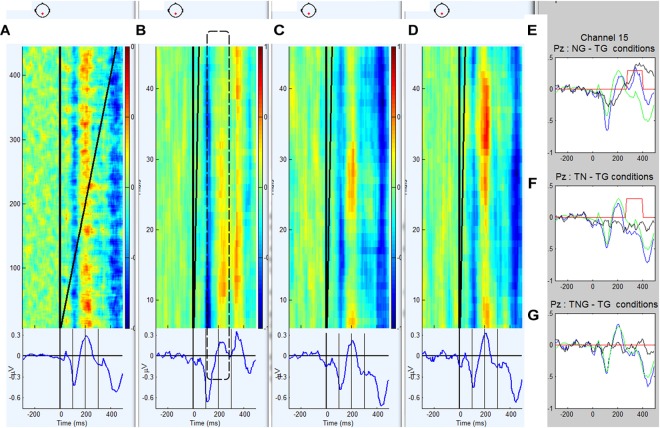
**(A–D)** Grand average ERP waveforms and trial by trial voltage plots at Pz electrode in 12 participants diagnosed with schizophrenia in the standard goal (TG), novel preceding goal (NG), novel target (TN) and simultaneous novel and goal (TNG) conditions. **(E–G)** Waveforms generated by subtraction (in black) of novel conditions from control condition (TG in green) and corresponding *t*-values for successive time bins of 187.5 ms.

Prior to the detailed analyses, the EEG data were averaged by condition to determine the latency ranges that would be best for estimating responses in single trial analyses. The grand average ERP waveforms associated with standard goal stimuli (TG), novel only (TN), simultaneous novel and goal (TNG) and novel preceding the goal (NG) conditions for the schizophrenic group and control group are shown in Figure 3, 4.

The waveforms were characterized by a positive peak between 200 and 250 ms after the first stimulus for conditions TG, TN, and TNG and 300 and 450 ms for condition NG. Therefore, in the NG condition, the P300 response to the preceding novel stimuli was estimated on a trial by trial basis as the maximum peak between 250 and 450 ms. In Figure 3 the across subject averaging for each trial in Pz electrode and weighted for Pz electrode shows in color the fluctuations trial by trial for each condition: TG, NG, TN, and TNG, respectively. From Figure 3, it is clear that NG is changing positively in the different trial averaging in the [250, 450] ms range clearly along the experiment, while TG, TN and TNG are not (see dashed line). Supplementary Material added statistical *t*-test difference between condition TG and each one of conditions NG, TN, and TNG (*p* = 0.001) and a window time of 187.5 ms and the comparison between both groups.

Both groups exhibit a significant P3 response to the novel stimuli that replace tone cue (in NG-TG condition) and this response is larger in the control group than the schizophrenic group (*p* = 0.01). This is consistent with previous research that suggests a reduction in the effectiveness of cognitive processes attributed to P300 in schizophrenia (e.g., Özgürdal et al., 2008; Kirihara et al., 2009), and also for P50 and N100 reduction (Terrasa et al., 2018).

The ERP difference TN-TG condition shows that the brain response of the controls is significantly more negative than that of the schizophrenics during the early part of the response to a novel stimulus that has replaced a goal stimulus. This suggests that the schizophrenic participants may be producing a smaller MMN to the novel S2 stimuli consistent with previous research auditory deviants in visual task in schizophrenic patients (Catts et al., 1995) and auditory deviant in auditory task in schizophrenic patients but not in bipolar and depressive patients (Umbricht et al., 2003).

### Single Trial Across-Subject Comparisons of P300 Amplitude and Intertrial Intervals for Novel Stimuli

**FIGURE 5:**
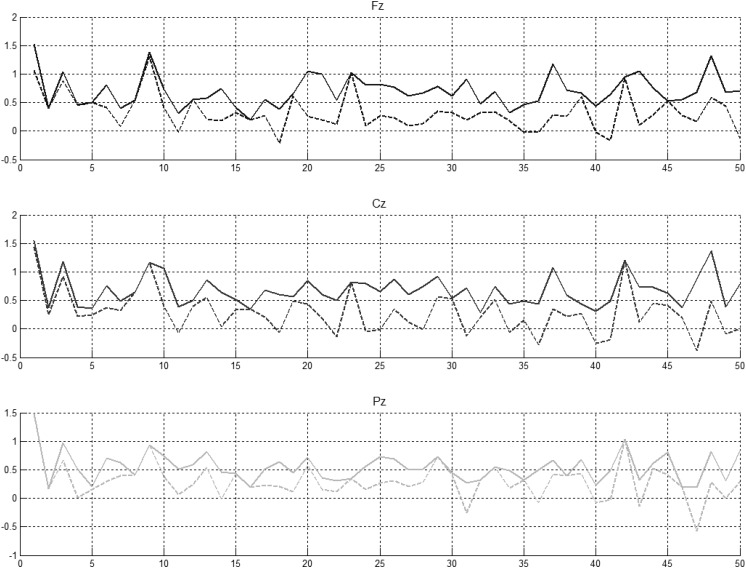
Preceding novel stimuli (NG) vs. amplitude of the P300 peak in Fz, Cz, and Pz. P300 peak amplitudes between 250 and 450 ms (solid lines) and between 350 and 450 ms (dotted lines) computed for control participants.

**FIGURE 6:**
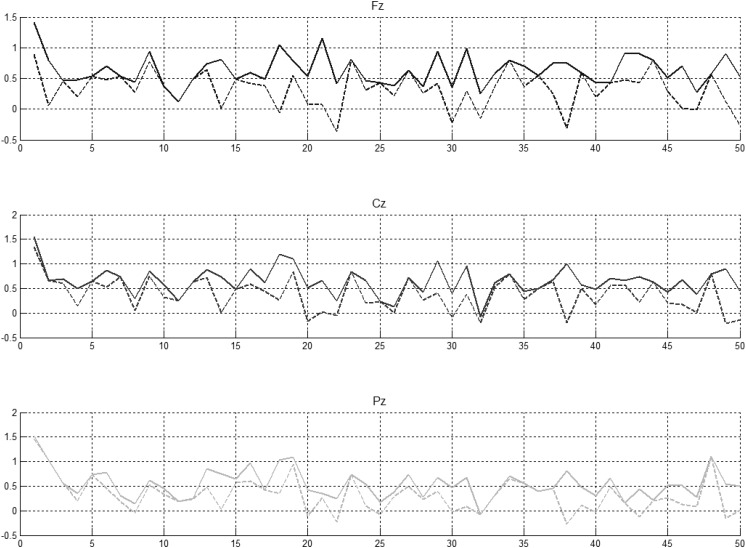
Preceding novel stimuli (NG) vs. amplitude of the P300 peak in Fz, Cz, and Pz. P300 peak amplitudes between 250 and 450 ms (solid lines) and between 350 and 450 ms (indented lines) computed for schizophrenic patients.

Peak amplitude of the EEG in the latency window 250–450 ms in each NG trial in the experiment was determined and is illustrated in Figure 5 for controls and Figure 6 for individuals with a diagnosis of schizophrenia. Independent sample *t*-tests were used to find whether the mean across-subject amplitude differed from NG trial to NG trial at Fz, Cz, and Pz.

It was evident that there were statistically significant differences between some pairs (Figure 6, left part) but little evidence of habituation of P300 amplitude over the time after the initial NG trial. When we arranged the number of trials between 2 preceding novel stimuli vs. amplitude of the P300 peak in Fz, Cz, and Pz (Figure 6, central part), no pattern of increase, decrease or oscillation of the amplitude of the P300 peak was found. A bootstrap correlation (1,000 random resamples) was run on data from channels Fz, Cz, Pz, CP6, and CP5, between the amplitude of the P300 peak and the number of trials between 2 preceding novel trials (Figure 6, right part) and a significant correlation of 0.4 was observed at the central electrode Cz.

In summary, it was found that that amplitude of P300 peak did not decrease over the duration of the experiment. Fluctuations in P300 amplitude were shown to be correlated with interval size between successive NG trials at Cz.

Peak amplitudes in five channels between 250 and 450 ms and between 350 and 450 ms were computed for both groups of controls and schizophrenic patients. Those amplitudes were correlated with the time between novels, bearing in mind the previous novel trial can be any of the TN, TNG, or NG conditions. Our analysis addressed two possibilities for the effect of time between novel stimuli defined by number of trials: (1) between the previous NG and the current NG; and (2) any novel (NG, TNG, or TN) that is the closest to the current NG (see Table 3).

**Table 3:** Correlations between peak amplitude in EEG channels Fz, Cz, Pz, CP5, and CP6 and time between Novels.

Controls (*n* = 20)						Schizophrenic patients (*n* = 12 )				
		
Peak between 250 and 450 ms. NG to next NG						Peak between 250 and 450 ms. NG to next NG				
		
Channel	*r*	*p*	CI1	CI2		Channel	*r*	*p*	CI1	CI2
Fz	0.211	0.150	-0.323	0.622		Fz	**0**.**279**	**0.019**	**-0.148**	**0.642**
Cz	-0.019	0.878	-0.473	0.471		Cz	0.182	0.100	-0.210	0.562
Pz	-0.083	0.589	-0.552	0.451		Pz	**0**.**307**	**0.007**	**-0.100**	**0.644**
CP5	-0.144	0.289	-0.539	0.348		CP5	0.148	0.230	-0.294	0.580
CP6	-0.040	0.801	-0.538	0.544		CP6	0.219	0.080	-0.223	0.607

**Peak between 350 and 450 ms. NG to next NG**						**Peak between 350 and 450 ms. NG to next NG**				
		
**Channel**	***r***	***p***	**CI1**	**CI2**		**Channel**	***r***	***p***	**CI1**	**CI2**

Fz	-0.087	0.662	-0.613	0.570		Fz	0.163	0.194	-0.278	0.589
Cz	0.053	0.763	-0.491	0.576		Cz	0.072	0.577	-0.391	0.500
Pz	0.128	0.425	-0.447	0.634		Pz	0.188	0.131	-0.259	0.615
CP5	0.048	0.731	-0.415	0.537		CP5	0.074	0.589	-0.448	0.556
CP6	-0.023	0.900	-0.507	0.524		CP6	0.254	0.049	-0.206	0.693

**Peak between 250 ms and 450 ms. Any novel to next NG**						**Peak between 250 ms and 450 ms. Any novel to next NG**				
		
**Channel**	***r***	***p***	**CI1**	**CI2**		**Channel**	***r***	***p***	**CI1**	**CI2**

Fz	-0.130	0.428	-0.605	0.471		Fz	-0.016	0.901	-0.520	0.455
Cz	-0.134	0.420	-0.689	0.411		Cz	-0.001	0.997	-0.510	0.479
Pz	-0.168	0.265	-0.621	0.370		Pz	0.032	0.818	-0.459	0.488
**CP5**	**-0.272**	**0.032**	**-0.625**	**0.208**		CP5	-0.118	0.300	-0.498	0.296
CP6	0.080	0.685	-0.503	0.602		CP6	-0.032	0.835	-0.478	0.517

**Peak between 350 and 450 ms. Any novel to next NG**						**Peak between 350 and 450 ms. Any novel to next NG**				
		
**Channel**	***r***	***p***	**CI1**	**CI2**		**Channel**	***r***	***p***	**CI1**	**CI2**

Fz	0.115	0.469	-0.480	0.560		Fz	0.014	0.908	-0.403	0.513
Cz	-0.064	0.674	-0.613	0.463		Cz	-0.083	0.561	-0.526	0.413
Pz	-0.013	0.913	-0.583	0.515		Pz	0.094	0.505	-0.362	0.561
CP5	-0.082	0.608	-0.617	0.490		CP5	-0.135	0.365	-0.590	0.419
CP6	0.090	0.594	-0.481	0.600		CP6	0.196	0.142	-0.278	0.620


*r, Bootstrap correlation. P, significance of the value of the bootstrap correlation. CI1, lower confidence interval value at 95%. CI2, lower confidence interval value at 95%. Statistical values (r, p, CI1 and CI2) were computed with 5000 resamples under bootstrap calculi. Significant values were highlighted in bold.*

We found that the P300 amplitude varied significantly with the ISI. In control participants correlations between any previous novel and the current NG condition and the peak amplitudes computed between 250 and 450 ms in controls was found significant in CP5 (*r* = -0.27, *p* = 0.0317, highlighted in Table 3). However, schizophrenic patients showed significant correlations in Fz and Pz (*r* = 0.27, *p* = 0.01915 in Fz and *r* = 0.30, *p* = 0.0067 in Pz, highlighted in Table 3) when correlations were computed between two NG conditions for peak amplitudes between 250 and 450 ms. No significant correlation difference was found in the other times, namely from 350 to 450 ms. Moreover, across electrodes with linear modeling was tested the influences of sound properties (see Supplementary Material).

### Single Trial Approach: Correlations Between Preceding Novel P3a Amplitudes and Stimuli Sequence and Sound Properties

The aim of this analysis was to dissociate P3a amplitude fluctuations that result from stimulus properties from group differences in attention orienting. Therefore, the correlations between preceding novel P3a amplitudes and stimulus sequence and the correlations between preceding novel P3a amplitudes and stimulus sequence were computed with *p* < 0.05. An analysis for the effects of sound measures including their relationship to preceding sounds in the design of the experiment demonstrated that sound properties did not differ between the sounds presented to the right and left ears (detailed results of these calculations are not presented here). The 50 preceding novel stimuli were split into two classes to analyze possible effects of stimulus differences. There were: 26 white noise stimuli with the same duration and few changes in amplitude, and 24 ‘environmental sound’ stimuli.

**FIGURE 7:**
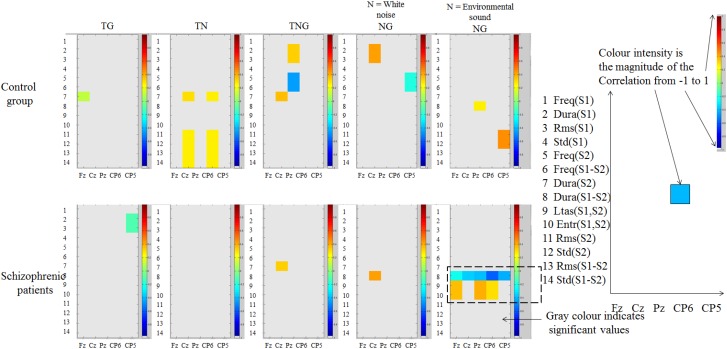
Correlations in control participants and schizophrenic patients (shown in color) between amplitude of single trial across-subject P300 peak at channels Fz, Cz, Pz, CP6, and CP5 (horizontal axis) and 14 sound properties (vertical axis). P300 amplitude measured in the time range [250 450] ms. Difference of duration and spectrum calculations (LTAS and entropy) showed correlations across electrodes in the analysis only in schizophrenic patients.

A 5,000-bootstrap correlation of sound properties of one or both stimuli (preceding novel and target number) with the across participant single trial EEG average in control (*n* = 20) and schizophrenic patient groups (*n* = 12) was computed. Table 4 illustrates these properties which consist of 14 measures computed from the current condition between the cue (preceding novel or tone) and target (goal/goal with novel/novel). In Figure 7 the amplitude of correlations between across-subject single trial P300 amplitude and the 14 stimulus properties (Table 4) are illustrated for each condition TG, TN, TNG, and NG considering when the Novel is either the white noise or the environmental sound. The magnitude of the correlation is indicated in color (see legend in Figure 7).

**Table 4:** Sound properties explored on the events of the experiment.

Stimuli name	Number of presentations
Freq(S1)	Frequency of S1
Dura(S1)	Duration of S1
Rms(S1)	Root mean square (RMS) in time of S1
Std(S1)	Standard deviation of S1
Freq(S2)	Frequency of S2
Freq(S1-S2)	Frequency of S1 – frequency of S2
Dura(S2)	Duration of S2
Dura(S1-S2)	Duration of S1 – duration of S2
Ltas(S1,S2)	Average difference in the long term average spectrum between S1 and S2
Entr(S1,S2)	Normalized mutual information in frequency between S1 and S2
Rms(S2)	Root mean square (RMS) in time of S2
Std(S2)	Standard deviation of S2
Rms(S1–S2)	Root mean square in time of S1 – Root mean square in time of S2
Std(S1–S2)	Standard deviation of S1 – standard deviation of S2


In the control group, in Figure 7 (top), the magnitude of the correlations is stronger at the parietal channel (Pz) in the simultaneous novel and goal conditions. This correlation is slightly stronger when either white noise or environmental sound is considered across these control participants. However, in control participants, the correlations between sound properties and P300 amplitude are not consistently spread across those five channels in this analysis (horizontally in Figure 7) and that means single electrodes activated on a specific time.

In the schizophrenic patients group, as shown in Figure 7 (bottom), the correlations in the first four conditions were not spread across electrodes or in the white noise condition. Unlike the control group, in the ‘environmental sounds’ the schizophrenic group showed significant correlations across at least three electrodes analyzed. In other words, for the schizophrenic group, when the warning signal is replaced by an environmental sound as a preceding novel distractor, the effect of duration of the sound is a significant negative correlation spread over all five channels of analysis. In contrast, the mutual information of frequency (LTAS) or entropy between S1 and S2 and the amplitude of P300 is strong and positive.

Due to the small sample size in both groups, correlations between groups are not possible to compare with Z-Fisher correlations. For example, when the Z-Fisher correlations in schizophrenic patients are between -0.3 and -0.6 and when the sample size (*n* = 12) is computed against *r* = 0 for control group (*n* = 20) this results in non-significant correlation differences.

A bootstrap correlation of previous properties of one/both stimuli (preceding novel and goal number) with the current EEG average in the task in control participants was also carried out, to explore why local probability and sound properties do not correlate with changes in P300 amplitude. Correlations between P300 amplitude over electrodes Fz, Cz, Pz, CP6, and CP5 and sound properties were computed for two ranges of time: [350, 450] ms and [250, 450] ms. To explore in more detail the nature of the correlations with the first 14 parameters used before, the 40 additional correlations described in Table 2 were computed separately for novel sounds presented to the left or right ear. Because of the 10 sound properties in the 4 additional comparisons, there are several groups of correlations. Bearing in mind whether white noise or environmental noise is analyzed and peak amplitude or peak latency four analyses may be done, the following was determined.

First, the correlations were computed between the sound properties of 26 white noise preceding novel stimuli and amplitudes of P300 (detailed results of these calculations are not presented here). This showed that left ear stimulation produces many significant and strong P3a correlations and many of them are correlated between the same sound pairs. This occurs across a wide range of computed sound properties and they are stronger in: Cz, Pz, CP6, and CP5 for sounds present on the left ear, Fz, Cz, Pz, and CP6 for sounds present on the right ear when the properties are related to previous novel sounds.

### Conditions and Stimulus Sequence Contextual on ERPs in Controls and Schizophrenic Patients’ Groups

The previous analyses indicated that there are correlations between several sound properties of the prior stimulus and the P300 amplitude. This was explored further by producing new averages of the control condition responses separated on the basis of which experimental condition the control trials followed computed with *p* < 0.05. This procedure rendered seven conditions: Tone-Goal preceded by Tone-Goal (TG.TG), Tone-Goal preceded by Tone-Novel (TN.TG), Tone-Goal preceded by Tone-simultaneous Novel/Goal (TNG.TG), Tone-Goal preceded by Novel-Goal (TG.NG), Tone-Novel preceded by Tone-Goal (TG.TN or TN), Tone-simultaneous Novel/Goal preceded by Tone-Goal (TG.TNG), Novel-Goal preceded by Tone-Goal (TG.NG).

**FIGURE 8:**
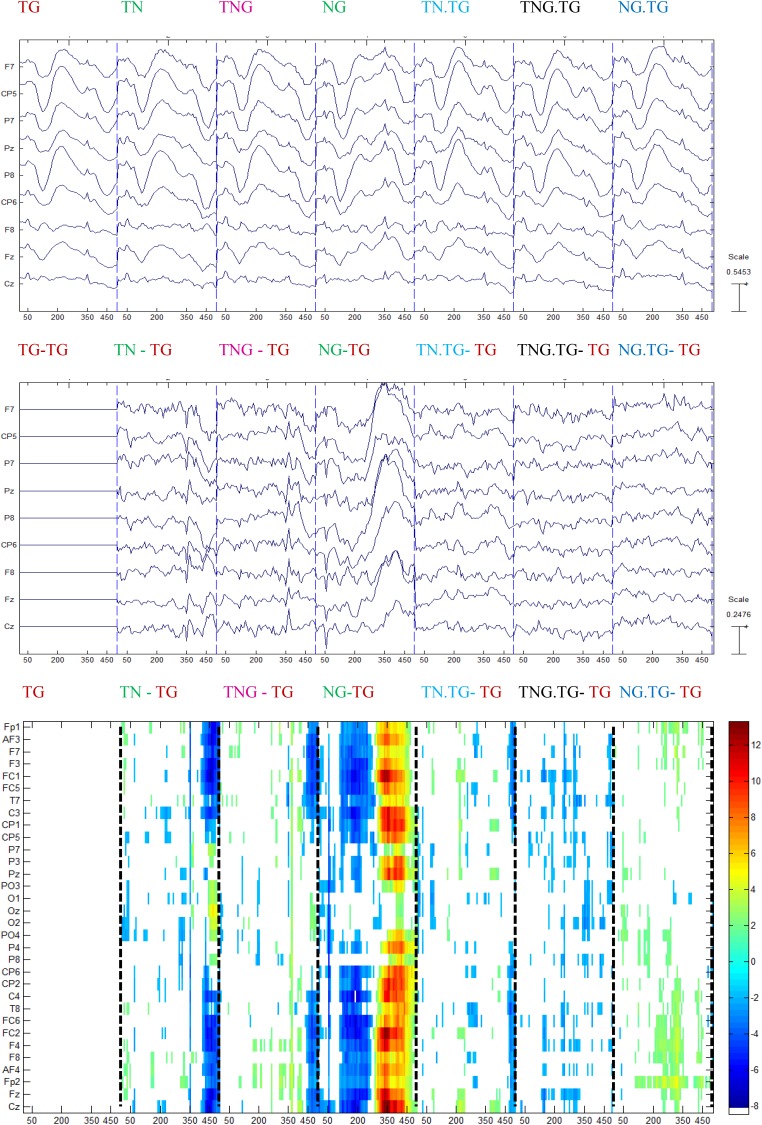
Grand average for control group of the ERP conditions (top) subtracted from every ERP condition in the previous channels (middle), and the one-tailed *t*-test analysis between each condition and the standard followed by the standard (*p* < 0.001) (bottom).

The control group showed significant differences, mainly in the range of time normally associated with perceptual and stimulus-driven processes. Figure 8 shows that the difference with the standard stimulus was not only for the other three different conditions (TN, TNG, and NG) but also when the condition of the preceding couple of sounds was considered (namely TN, TNG, and NG). The standard ERP was subtracted from the other ERP conditions to emphasize the differences between conditions (Figure 8, middle). Finally, multiple one-tailed *t*-tests between each condition and the standard condition were calculated (*p* < 0.001, uncorrected) to determine the significant differences in time and across channels (Figure 8, bottom). Significant differences are shown in TN-TG, TNG-TG and NG-TG as expected. These differences were stronger in the [200, 350] ms range of ERP difference NG.TG was shown to be significantly different from TG.TG mostly at the right lateralized electrodes (see Figure 8, bottom in dashed lines). Bearing in mind the ERP answer on the electrodes on the top, it may suggest a kind of positivity response for S1 and the P50 for S2.

**FIGURE 9:**
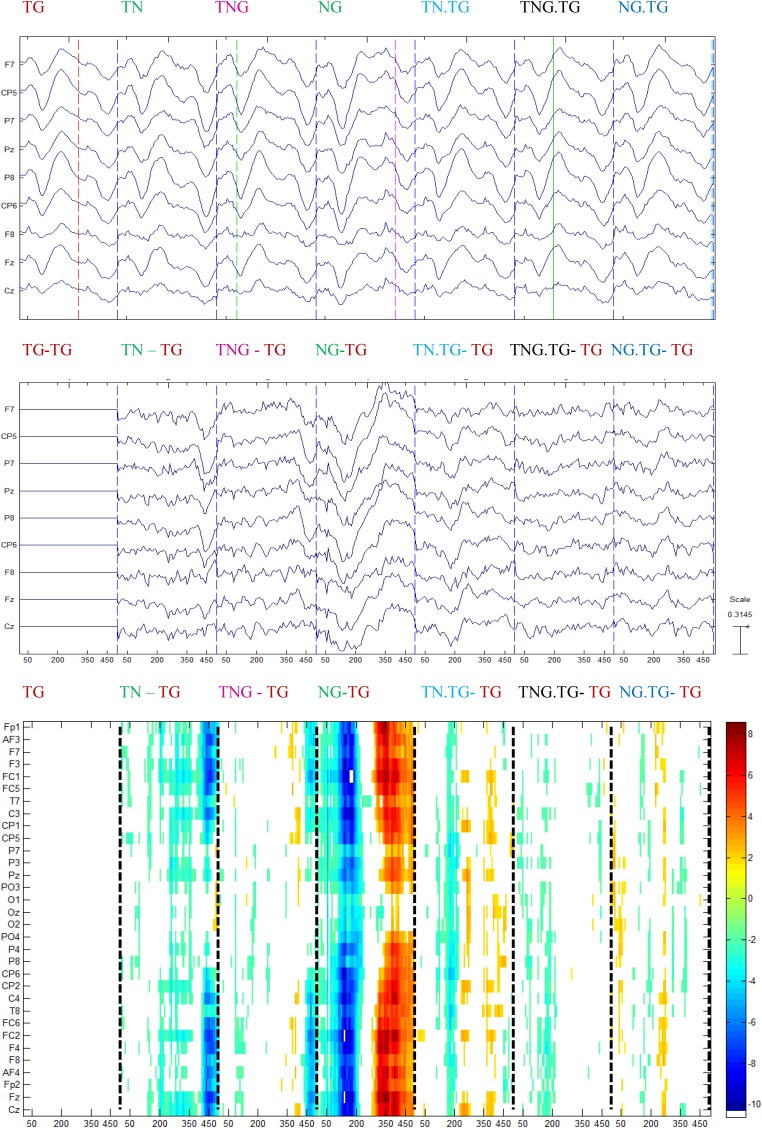
Grand average for schizophrenic patients of the ERP in each condition (top) subtracted with the standard ERP condition in the previous channels **(middle)**, and the multiple *t*-test analysis between each condition and the standard followed by the standard (*p* < 0.001) (bottom).

In the case of schizophrenic patients, significant differences occurred at the time that can be attributed to gating of sounds (P50) either in the first or second stimulus. This is shown in the NG – TG plot in Figure 9. Similar to control participants, Figure 9 shows that the difference with the standard stimulus was not only for both different conditions but also in the standard condition split into those four conditions relying on the condition of the preceding couple of sounds.

The schizophrenic patients showed significant differences, mainly in the range of time normally associated with perceptual and stimulus-driven processes. Figure 9 shows that the difference with the standard stimulus was not only for the other three different conditions (TN, TNG, and NG) but also when the condition of the preceding couple of sounds was considered (namely TN, TNG, and NG). The standard ERP was subtracted from the other ERP conditions to emphasize the differences between conditions (Figure 9, middle). Finally, multiple one-tailed *t*-tests between each condition and the standard condition were calculated (*p* < 0.001, uncorrected) to determine the significant differences in time and across channels (Figure 9, bottom). Significant differences are shown in TN-TG, TNG-TG, and NG-TG as expected by the impairment hypothesis (H3). Bearing in mind the time range of more than 50 ms of difference, the NG.TG was not shown to be significant different from TG; instead, TN.TG and TNG.TG were different.

Overall, it was found that the sequence effects in contextual sorted ERPs indicated a difference in these groups. Whether in control and schizophrenic patients, the previous stimulus significantly affected the following standard condition ERP deflections.

## Discussion

Currently, it is believed that P300 deflections consist of a P3a related to attention activation and P3b related to context-updating operations and memory storage (Polich, 2007). Here, we have found ERP evidence of differences in the distribution of the P3a component, which suggests a dissociation of activity in the SDN and GDN of the attention reorienting system (Corbetta et al., 2008). In the present reanalysis of data from a group of individuals with schizophrenia and a group of healthy controls, the results suggested that ERP deflections are significantly influenced not just by the probability of the stimulus type (not supporting H1) but also by trial by trial differences in the frequency, duration and amplitude of the sounds (supporting H2). This analysis determined that different regressors in each group in response to these other factors would improve the specificity and/or sensitivity of the ERP analyses not only in P300 but also for MMN in schizophrenic patients (supporting H3). In summary, the original hypothesis H3 is confirmed with the reduction of MMN in controls and the tendency of the greater reduction of MMN the larger in time of the Novel for schizophrenic patients. The larger the mutual frequency information is between S1 and S2 the larger the P300 in the case of Schizophrenic patients, but not in the case of the controls SDN attenuation as this may be a consequence of stimulus properties for the multiple condition task.

### Behavioral Results

When mean RTs were subjected to statistical analysis, there was more slowing of RT in the preceding novel condition (NG) than in the simultaneous novel and goal (TNG) condition, suggesting that attention orienting occurred in the NG condition and involved a temporary shift in the mental representation of the auditory scene. Although the RTs of the schizophrenic group were significantly slower, there was no interaction between Group and Condition. The basis of these differences was explored further by carrying out a running average analysis of individual participants and it was observed that only 15 out of 20 control participants demonstrated a consistent distraction effect.

### ERP Results: Novelty Distractor Informational Content and Stimulus Probability (H1)

The results showed that Novel P3a amplitude showed significant variation over time but did not decrease in the long-term and was not simply predicted by inter-trial intervals as predicted by Gonsalvez and Polich (2002) with small and non-significant correlations in the control participants but with significant correlations in the schizophrenic patients (around *r* = 0.3 in Fz and Pz).

The findings of P300 with significant variation with ISI, defined differently in both control and schizophrenic patient groups, can reflect a different processing in this particular task. On the one hand, controls showed significant correlation to the left side (*r* = -0.27, *p* = 0.03 in CP5); this would be consistent with attention to a known task (Corbetta and Shulman, 2002). On the other hand, schizophrenic patients showed significant correlations in frontal and parietal electrodes (*r* = 0.27, *p* = 0.02 in Fz and *r* = 0.30, *p* = 0.0067 in Pz) which may be correlated with orienting of attention (Gonsalvez and Polich, 2002).

**FIGURE 10:**
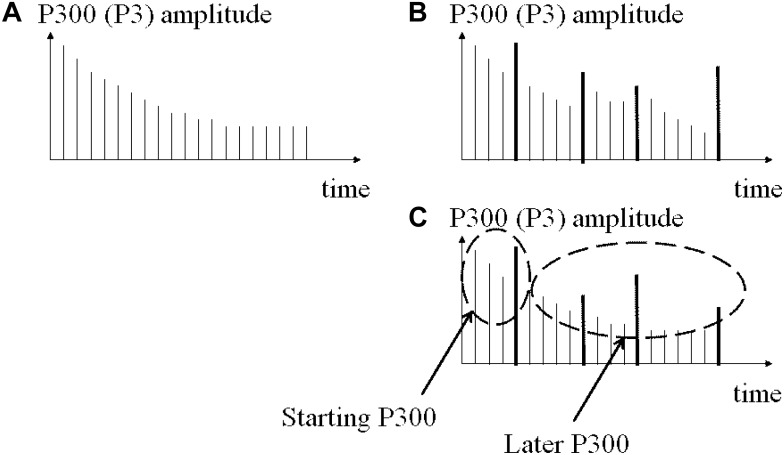
Initial hypothesis plotted with the first results and the route to the sound properties analysis. **(A)** Theory of habituation response to stimulus sequence. **(B)** Initial hypothesis about time dependence of novel amplitude. Found only in some left electrodes in controls. **(C)** Most of the amplitude channels explained by significant correlations with stimulus properties in both groups.

Therefore, with reference to Figure 10, the findings do not fully support the first hypothesis (illustrated in Figure 10) that the larger the time between two novel stimuli the larger the P300 (H1). In other words, given H1 as it is drawn in Figure 10A,B, the results show: negative correlated effects in the left hemisphere in control participants, pointing to an unexpected electrical behavior in Figure 10C, and a positive correlated novelty effect in frontal and parietal electrodes in schizophrenic patients, pointing to an electrical behavior in Figure 10B.

A possible explanation is that the four different conditions produce different processing outcomes. In this way, in both groups the P300 response to novel stimulus show different evidence of processing novel and different conditions in the left hemisphere for the longer the time duration between two NG conditions; this suggests that the time between conditions is producing an alerting effect in controls. There is also evidence of frontal and parietal electrodes answering positively to the longer time duration between two novel conditions which suggests prefrontal scalp control and having different parietal electrodes measures and producing reorienting of attention in schizophrenic patients.

Barbalat and colleagues employed structural equation modeling in the participant responses to a letter discrimination paradigm using a first cue as the episodic signal and a contextual signal to decide the finger answer to the task. They found impairment in the connectivity of the dorsolateral prefrontal cortex for schizophrenic patients (Barbalat et al., 2011). Using functional connectivity for the parietal cortex and the prefrontal cortex (PFC), Tan and colleagues, in a N-back memory task, found that connectivity was greater in the schizophrenic patients for ventral PFC and greater in the control group for the dorsal PFC (Tan et al., 2006). Although scalp EEG does not inform about brain source, regarding the results in the present experiment in the Fz electrode, the group differences may be explained by a different interaction of P3 with the inter-stimulus effects which made it difficult to identify a clear pattern of increase or decrease in P3 amplitude as the number of preceding stimuli increase. In addition to that, the control participant at left parietal electrode CP5 and the schizophrenic patient central parietal electrode at Pz electrode may be the subject of reanalysis in other functional Magnetic Resonance Imaging (fMRI) studies, for example, in Barbalat et al. (2011) experiment, parietal regions were not explored.

In addition, in a behavioral experiment using novel sounds in a visual categorization task, Parmentier and colleagues found that behavioral distraction depended on the informational value of the sound changed. They claimed that the low probability of occurrence of a novel sound did not constitute a sufficient condition for behavioral distraction (Parmentier et al., 2010). In this way, it would be inaccurate to assume that an auditory novel event elicits distraction due to its low base rate probability. We showed this in behavioral (alerting and non-facilitative RTs) and ERP results having stimulus properties correlated with P300 in different Novel properties at different conditions. Our current findings with ERPs associated with orienting of attention at P3a in the preceding novel condition complement their idea, including the properties of stimulus and condition task switching.

Following the route that the less expected (in time) the stimulus the larger the amplitudes on ERPs (Squires et al., 1976), we can keep/update that phrase saying that the less expected the stimulus (i.e., differences between the current stimulus and a previous stimulus or by the larger inter stimulus intervals) the larger the ERP amplitudes (see Figure 10).

### Stimulus Sequence Effects vs. Stimulus Properties (H2)

Using an innovative method for stimulus properties extraction features and EEG analysis, we found that P3a amplitude showed correlations of different magnitude in the range 0.3–0.6. This was dependent on whether stimuli were presented to the left or right ear for the different properties based on Sound Duration, Mean Amplitude and Frequency.

A correlation was found between P3a (measured after onset time from 350 to 450 ms) and the durations of previous sound stimuli. However, the results in this experiment showed significant correlations with previous sound durations in novel sounds that are linked to the frequency and amplitude of the sounds. Therefore, the second hypothesis (H2) is supported for frequency and amplitude but not systematically for duration because of these confounding interactions.

**FIGURE 11:**
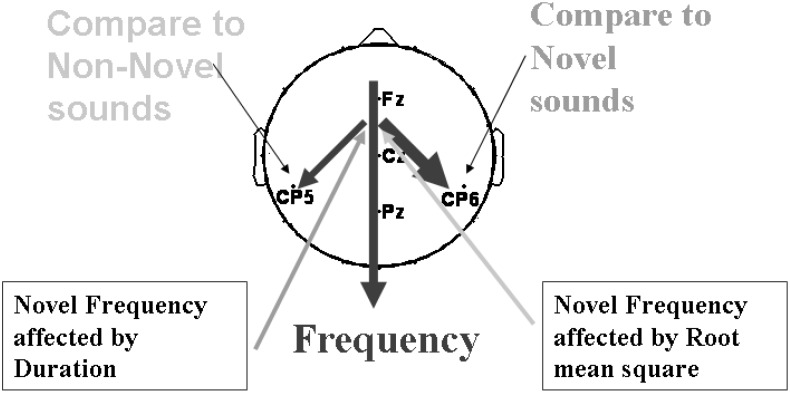
A general route of the sound properties analysis influencing P3a amplitude. Thickness shows strength of the correlations found.

Figure 11 suggested a model that, when the current sound is compared with previous Non-novel sounds, then correlations are strongest in the left hemisphere, and when the current trial is preceded by a novel trial then correlations are stronger in the right hemisphere.

Contextual stimulus properties had significant influences on P3 amplitude in both control and schizophrenic patients. On the one hand, in the control group this is mainly in the stimulus-driven and perception time (0–300 ms) between conditions in standard condition. On the other hand, schizophrenic patients showed differences in the range of time of gating sounds, P50 either in the first or second stimulus between conditions and within standard condition as well.

Liao et al. (2016) employed properties at two different frequencies at 1 and 2 kHz and were successful in dilating pupils at 2 kHz (oddball) and noise. In our work, we employed Parmentier et al. (2010) as a baseline in the discussion because of the different tests done in that article in regard to stimulus probabilities and stimulus durations that affected RTs. Parmentier and colleagues claimed that the advantage of the cross-modal oddball task shows the primordial role of the sound’s informational content as demonstrated by the finding of a facilitation of performance by novels when these predicted with certainty the occurrence and timing of targets while standards did not (Parmentier et al., 2010). A recent report suggested that visual distracters over auditory stimulus would require less trials to evoke distraction (Córdova Berríos et al., 2018). However, in our purely experimental auditory results, when sound was stripped of its informational value, auditory novelty had an impact on ERP waves and this indicated that the late brain processes also have the informational content of the previous experience.

Parmentier et al. (2010) also indicated that behavioral distraction following a novel or deviant sound reflected a delay in the processing of the target, as the consequence of time penalties associated with the shift of attention only operate within the bounds of a goal-relevant stream of auditory events. Our study suggests that in controls, this involves the SDN as well. This can be generalized by any change of either cue or target that would reflect a different brain process.

Parmentier et al. (2010) suggested that behavioral distraction measured in the cross-modal oddball task is only observed when the irrelevant sound presented to participants provided useful information regarding the upcoming task-relevant stimuli. When stripped of this information, novel sounds produced no distraction. In this study, based on stimulus duration effects, we believe that the properties of the sounds are relevant for the ERP response when the significance of the inter-stimulus properties is changed. Specifically, in the present experiment, the inter-stimulus properties were not significant in several conditions that switched attention in several ways and this shows that stimulus properties are significant information of the upcoming stimuli.

In the general linear modeling approach, the Second Level Analysis based on Two-samples *t*-test for group comparison reported differences between TNG and NG conditions. The main differences were larger ERP deflection for controls in MMN and P300 for NG condition and smaller in the TNG condition. Also, the R2 values found in the first level analysis and the different regressors found in each condition suggests that the task involves more than a simple activation of stimulus-driven and goal-driven attention networks. Limitations: It is important to point out that this analysis has the following limitations: on the one hand, in the accuracy of connectivity of bins because of the number of channels (32) and the sampling frequency (128 Hz); and on the other hand, in several frequency properties estimated from the task (detailed results of these calculations are not presented here) as well as in our non-parametric design, which produces variable R2 value distributions across participants. These limitations may result in the smaller correlations measured in some participants.

These sets of regressors coming from both correlation analysis and general linear modeling in EEG data can be explained taking into account:(1) episodic memory; (2) contextual control; or even more significantly (3) attention to details in our attention paradigm design. Limitations: The experiment was carried out with an imbalanced group number *N* = 20 for controls and *N* = 12 for schizophrenic patients, although LIMO (see Supplementary Material) provided a multi-comparison this difference limited the comparison between groups.

### Effect of the Immediately Previous Trial Context on Current Attention (H3)

**FIGURE 12:**
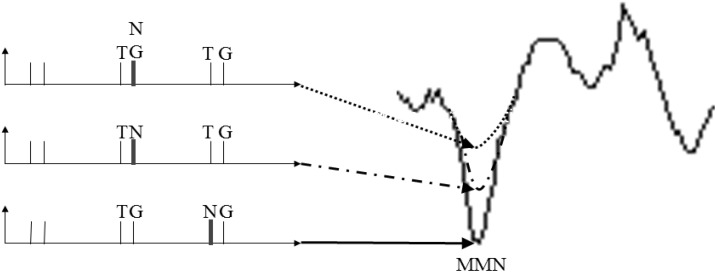
The context interpretation about MisMatch Negativity in schizophrenic patients.

In both controls and schizophrenic patients, in Section “Conditions and Stimulus Sequence Contextual on ERPs in Controls and Schizophrenic Patients’ Groups”, it was observed that the previous stimulus affects the following standard condition ERP deflections. In the control group, ERP deflections were found mainly in the stimulus-driven and perception time (0–350 ms) for S1 and P50 for S2 at NG condition followed by TG condition. In schizophrenic patients, deflections were significantly different in gating sounds, P50 either in the first or second stimulus. Models of cognitive dysfunction in schizophrenia patients are frequently discussed as “stimulus-driven” versus “goal-driven” (reviewed by Javitt, 2009). The present findings based on previous trial context suggest that both types of dysfunctions are simultaneously present in schizophrenia extending the view of Leitman et al. (2010) to the temporal scale. Explaining in detail when the immediately previous context is considered in terms of MMN, it was found that the trial pair NG.TG produced a larger MMN, followed by TN.TG and TNG.TG (see Figure 12). Our interpretation is that the novel causes a smaller MMN when the novel is before the cueing effect (TN in dashed and dotted curve) and even less when either is mixed with the goal or having half of the power (TNG in dotted curve). Therefore, this context-dependent interpretation has two supporting literature findings: (1) it is consistent with the lower amplitude MMN (NG.TG, TN.TG and TNG.TG) or longer latency in MMN peak proposed in a review by Javitt (2000); and (2) it complements results in the case of a sort of different time presentation (300, 1,500, and 1,500 ms respectively adding a 2,150 ms for NG.TG) resulting in different sensory deficit in schizophrenia patients in the results of auditory MMN. This may be explained using distributed hierarchical models for deviant stimuli in MMN (Leitman et al., 2010). The results may therefore be consistent with different neurochemical theories of the effects of schizophrenia on MMN, considering *N*-Methyl-D-aspartate (NMDA) antagonists (Javitt, 2000; Heekeren et al., 2008) and the serotonin receptor (5HT2A) as an agonist giving a model of psychoses that display distinct neurocognitive profiles (Heekeren et al., 2008). Bearing in mind the route for attention and possible network interactions and adding the model for schizophrenia proposed by Ferrarelli and Tononi (2011), it will be interesting to explore techniques such as LORETA to study hierarchical modeling.

According to Baldeweg et al. (2002), frontal and central electrodes should show MMN attenuation. A simulation of an MMN experiment using predictive coding (Friston, 2005) and a hierarchical model of the brain based on relative changes on the task (Friston, 2008) showed the reduction of MMN in tone repetition in an auditory task (Moran et al., 2013). Our study complements this statement because TN.TG – TG has shown differences across several electrodes in both hemispheres and TNG.TG – TG appears mainly in the right hemisphere in the MMN. These suggest that the Goal stimulus is being processed in the left hemisphere and that attenuates the MMN difference and suppresses P300 differences.

Therefore, with regard to the third hypothesis (H3), H3 is supported and the larger the MMN the larger the P3a, but we also found an effect in time of the novel before the warning signal (S1) in the analysis for schizophrenic patients. In this way, when we have the tone as a cue, it is important if the previous sound was a novel or a novel simultaneously with the target. This interpretation suggests that these trial context effects should be explored further to determine whether the time is related with background stimulus for schizophrenic patients. Although scalp EEG does not provide unambiguous information about brain activity sources, this result is consistent with the idea that frontal lobe (shown in frontal channels) activity generates P3a, having in mind an impairment in processing the stimulus (Uhlhaas and Singer, 2006). In this way, the negative correlation of the distinction of two contiguous stimuli shown in schizophrenic patients at the beginning of Section 2.3.5 with stimulus properties can be studied with the progressive MMN reduction showed in this part. Finally, this is linked with the studies by Özgürdal and colleagues. They explored differences between controls, chronic schizophrenics and participants with first episode. Their results pointed to significant differences in those three groups in Pz electrode and the range of time to find the P300 peak was between 280 and 600 ms (Özgürdal et al., 2008). This is consistent with the time property found here, that is first episode participants are developing the time property MMN reduction and consequently a P300 reduction.

Gilmore and colleagues demonstrated that amplitude reduction of P3 in externalizing disorders was not affected by stimulus sequence effects. They found, as expected, that the greater number of standards preceding the target the greater P3 amplitude. Sequence effects in amplitude reduction of P3 were found normal in externalizing disorders and they suggested that such individuals are able to effectively utilize context during the oddball task to form subjective expectancies about the probability of a target occurring (Gilmore et al., 2012). Limitations were suggested coupling N200 and P3 with regards to stimulus sequence (Harper et al., 2016); however, we found that control and schizophrenic patients show P3 amplitude changes modulated by stimulus properties and contextual effects, but one needs to carefully interpret the present results because of the four conditions presented in the task and the same stimulus sequence for each participant.

### Mutual Information Is a Covariate for Schizophrenic Patients

In the five channels of analysis (Fz, Cz, Pz, CP6, and CP5), we found that the correlation between P300 and mutual information in the frequency domain, under a cue and orienting mixed auditory paradigm, evokes a right lateralized significant P3 amplitude reduction in schizophrenic patients. With this we have shown that the purely auditory oddball task allows studying informational content. Parmentier and colleagues claimed that in an auditory oddball task, the distracter and the target are embedded into the task and this does not allow the independent manipulation of the distracter’s informational content (Parmentier et al., 2010). We can re-state their claim and go further: when the distracter information is shared with the goal, this sharing can control the P300 wave, the biomarker of orienting response. This claim was shown in the schizophrenic patients where the greater the LTAS the greater the P300 response and in the control participants with the LTAS where the correlations considered the left sound lateralisation, as part of the results of the innovative analysis method. As such, it would be interesting to test this for the conflict monitoring task of the experiment, thus in the simultaneous novel and goal condition, and test if single trial correlation across several channels or a second level analysis in the general LIMO approach would validate or invalidate this informational content argument. Another interesting approach would be to insert novel (S1) followed by the simultaneous novel and target (S2) as a fifth condition.

Hughes and colleagues showed that the voice deviants were producing a disruption of the ability to identify the item from a standard set of items. This was reflected in variations in the RTs as evidence of behavioral distraction to deviant background items (Hughes et al., 2007). These findings were consistent with a previous study where a temporal deviation in ISI was used rather than a voice deviation (Hughes et al., 2005). These results were interpreted as support for a dual mechanism changing-state and deviation model. In the present experiment, correlations of current preceding novel condition (NG) were tested with the other previous conditions. Several correlations were particularly strong with other previous conditions. One can say therefore that in the cross-modal task, e.g., Hughes et al. (2005, 2007) or Parmentier et al. (2010), auditory distraction can be explained by the nature of the sound and the nature of the processing required in the task. Further, one can say that the ISI changes introduce differences in the processing of background stimulus.

From the point of view of the theory of mind in perceptual and attentional processes, the reduced ability to distinguish externally generated stimuli can be reflected by auditory hallucinations. According to Hugdahl, these auditory hallucinations are supported by thalamocortical sensory pathways, from internally generated inputs, which are processed by corticothalamic circuits (Hugdahl, 2009). The contextual effects of previous stimulus properties suggest that P50 gating is different in schizophrenic patients; therefore, a strong influence of thalamocortical activation should be implied in this process. The correlations between P300 and S1 durations were stronger in the right hemisphere, consistent with the right lateralized areas involved in reorienting of attention. In addition, in dichotic listening experiments, it has been shown that patients with schizophrenia have problems reporting the right ear stimulus (Green et al., 1994; Løberg et al., 2004). Therefore, we suggest that the mutual information that appears correlated with P300 amplitude in the stimulus-driven attentional network can reflect a different computation for schizophrenic patients. Assuming that in many schizophrenic patients there is an increased likelihood of auditory hallucination, schizophrenics are said to be in a state of hypervigilance and enhanced stimulus-driven processing to compensate for this impairment.

## Author Contributions

CM-V made the substantial contribution to the conception of the work, i.e., adapted paradigm for number, made the analysis and main interpretation of data for the work, drafted the work or revising it critically for most of the intellectual content and made final revision of the intellectual content. DP made the substantial contribution to the design of the work, he made the preliminary analysis and interpretation of data for the work, made data collection, and revised the manuscript critically for most of the intellectual content.

## Conflict of Interest Statement

The authors declare that the research was conducted in the absence of any commercial or financial relationships that could be construed as a potential conflict of interest.
