# Supplementary material for: Context Dependence Signature, Stimulus Properties and Stimulus Probability as Predictors of ERP Amplitude Variability
Source: Front Hum Neurosci. 2019 Feb 26;13:39. doi: 10.3389/fnhum.2019.00039 (PMC6399205; doi:10.3389/fnhum.2019.00039)
Supplement: Supplementary file 1 [file Data_Sheet_1.DOC]

Supplementary Material

# Context Dependence Signature, Stimulus Properties and Stimulus Probability as Predictors of ERP Amplitude Variability

Carlos Mugruza-Vassallo ^1,2*^, Douglas Potter ^2^

^1^Computing and Cognitive Neuroscience Laboratory, Engineering and Management, Universidad Nacional Tecnológica de Lima Sur, Lima, Peru

^2^Neuroscience and Development Group, Arts and Science, University of Dundee, Dundee, United Kingdom

*** Correspondence:**Corresponding Author
cmugruza@yahoo.com

# Supplementary Methods

In the third approach of EEG analyses, using sounds properties that influenced the P300 found in the first approach, all the conditions were next analysed using LIMO EEG (Pernet et al., 2011). For each subject, an ANCOVA model was used: the 4 conditions plus 2 covariates coding for sounds: LTAS and RMS between the current preceding novel (NG) trial and the previous NG trial, using a simple hierarchical model of the EEG data with βi as the constant and Si y Aj-8 as the categorical and the continuos regresors following the equation:

EEG = β0 + ∑ βiSi + ∑ βjAj-8 + Error

At the group level, Supplementary Figure 1 shows that a repeated measure ANOVA was conducted on the parameters computed for each condition. Because sounds properties that influenced the P300 were regressed out for each subject / trial, differences between conditions can only reflect differences due to novelty and not differences between stimuli in the different conditions. The purpose of this analysis was to explore sources of variability of P3a deflection associated with attention orienting.

# Supplementary Results

In an attempt to improve the explanation of the single trial averaging results from P300 amplitudes to the whole ERP waveform a new analysis was carried out using LInear MOdelling (LIMO) for EEG data (Pernet et al., 2011). This is based on the strongest significant values for Percentage of the variance (R2). For each subject, a design matrix was created that included the experimental conditions as categorical variables. LIMO was run for all conditions and R2 values were less than 0.15 for every participant.

First, both groups and the four conditions in a 2 x 4 ANOVA were explored. The F values for each electrode in each time bin are illustrated in Supplementary Figure 2. Comparisons of the Difference between conditions revealed a significant difference in the ranges of 0 to 50 ms and from 100 ms to 150 ms (stimulus detection), and also in the time window 184.38 ms to 215.63 ms (range between perception and mismatch negativity).

Bearing in mind the previous results, the multiple one-sample t-test was run in each group to explore in each condition two issues: first, to determine the significance of the ERP wave differences, and also by using the regressor in the analysis. Second, a multiple Two-sample T test was run in order to find the differences between Control participants and Schizophrenic patients.

In the TG condition for control participants and schizophrenic patients, the T-values based on time per electrodes using LIMO were done in each group. Results of multiple one-tailed T test reported similar deflections for both groups positive deflections in the ranges from 50 to 100 ms (stimulus detection) and from 170 ms to 270 ms and negative waves in the time window from 90 ms to 140 ms (range between perception and mismatch negativity) and from 300 ms (controls) and from 320 ms (schizophrenic patients). This confirms that the tone does not produce a significant P300 response, but the tone produces a strong response around 220 ms (controls) and from 200 ms (schizophrenic patients). When the difference between both groups was calculated, this revealed right lateralized differences between 86 and 156 ms (detailed results of these calculations are not presented here). Therefore, these results suggest a difference in the N100 response. There is also a small window time of difference at 351 ms. The greatest T-values were around 3.

In the TN condition for control participants and schizophrenic patients, computation of the T-values based on time per electrodes using LIMO were done in each group. Results of ERP significance show a small positive deflection lateralized from 180 to 260 ms and negative deflections in the time from 300 ms. Negative values are from 380 ms and negative deflections are stronger from 420 ms to 460 ms (controls) and from 400 ms to 470 ms (schizophrenic patients). Again, this confirms that the Tone does not produce a significant P300 response, and the possibility that the MMN of the Novel in S2 delays the negative deflection when the result is compared with TG condition. When the difference between both groups was run using bootstrap analysis (detailed results of these calculations are not presented here), this revealed right lateralized differences between 0 and 39 ms. Therefore, the results suggest a different auditory gating response in the first 39 ms, although the greatest T-values were around 3.

In the TNG condition for control participants and schizophrenic patients, the T-values based on time per electrodes using LIMO were done in each group.. Results of multiple one T test show right lateralized positive deflections from 180 to 260 ms (controls) and from 190 ms to 220 ms (schizophrenic patients) and negative waves from 300 ms being stronger from 430 ms to 470 ms (controls) and from 400 ms to 470 ms (schizophrenic patients). This negative deflection is possibly the MMN of the simultaneous Novel and Goal in S2. Again, this confirms that the Tone does not produce a significant P300 response, but it produces a maximum positive response at 226.6 ms for controls (absolute t-values greater than 4). When the difference between both groups was run (detailed results of these calculations are not presented here, absolute t-values close to 4) differences at 190 ms and at 398 ms appeared, although at 398 ms the difference were spread over the scalp, the results revealed a right lateralized differences between 300 ms and 440 ms. This suggest a different processing of the second stimulus for schizophrenic patients when the Novel and Goal were presented simultaneously.

In the NG condition for control participants, results of multiple one-tailed T tests show right negative values from 100 to 200 ms (absolute t-values greater than 6) and positive values from 280 ms and being stronger from 340 ms to 390 ms in controls and from 340 ms to 480 ms in schizophrenic participants (absolute t-values greater than 6). Different to the previous 3 conditions, this result confirms that the Novel produces a significant P300 response, and this is in spite of the Goal coming at 300 ms. In the comparison of the NG condition, Supplementary Figure 3 shows the T-values based on time per electrodes. Results of multiple Two-sample T-test positive waves in the time window from 20 ms to 40 ms and right lateralized 70 ms to 140 ms (range between perception) and a small bilateral positive deflection in the parietal electrodes from 398 to 430 ms. This confirms that between groups P300 is slightly different around 410 ms. Also, P50, and MMN is different for both groups. This finding would be consistent with difference in perceptual stage.

In the LTAS(S1, S2) regressor for control participants, long term average spectrum between S1 and S2, we seek for ERP significance in all trials. Therefore, Supplementary Figure 4 shows the T-values based on time per electrodes. Results of multiple one T test show right negative values from 100 to 200 ms (absolute t-values greater than 6) and positive values from 180 ms to 240 ms (absolute t-values greater than 6) and continuing up to 300 ms. This confirms that the Novel produces significant positive changes in N200 or MMN and P300 responses, and this is in spite of the Goal coming at 300 ms and also the magnitude of the T-values are similar to the magnitude of the TG condition.

The previous analysis was extended using the stimulus properties as the continuous regressors in the first level analysis. This is based on the strongest significant values for Percentage of the variance (R2). For each subject, a design matrix was created that included the experimental conditions as categorical regressors and the sound properties as the continuous regressors. In an attempt to distinguish group sources of variance from variance introduced by stimulus variables, every single condition was run added as covariates: firstly, each one of the sound properties (see Supplementary Figure 4), and secondly, pairs of sound properties in all the possible combinations worked in previous sections (Table 2). The addition of every sound property as the continuous regressor improves R2 values in several cases. Results relating to stimulus properties are sparse. Another finding from these plots is when one looks at those stimulus properties which are not predicting the variance of the data. For example, the stimulus properties 25, 27, 31, 32, 46, 55 and 56 are not making more R2 explanation than the conditions. More interestingly, the stimulus property 50 (Entr(S1R,S1(PN)R) (see dashed line) has a good explanation of the variance in several Control participants (R2 > .3) but not in more the half of the Schizophrenic patients (R2 < .2).

# Supplementary Figures and Tables

## Supplementary Figures


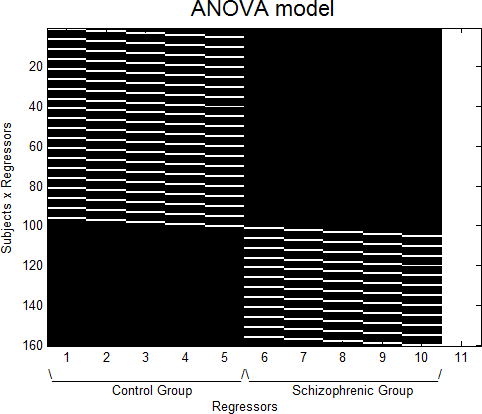


**Supplementary Figure 1.** Design matrix of the second level ANOVA model considering 4 conditions and 1 covariate in the first level analysis and taking these ones as 5 regressors in the second level analysis to make comparison between Control and Schizophrenic patients groups.


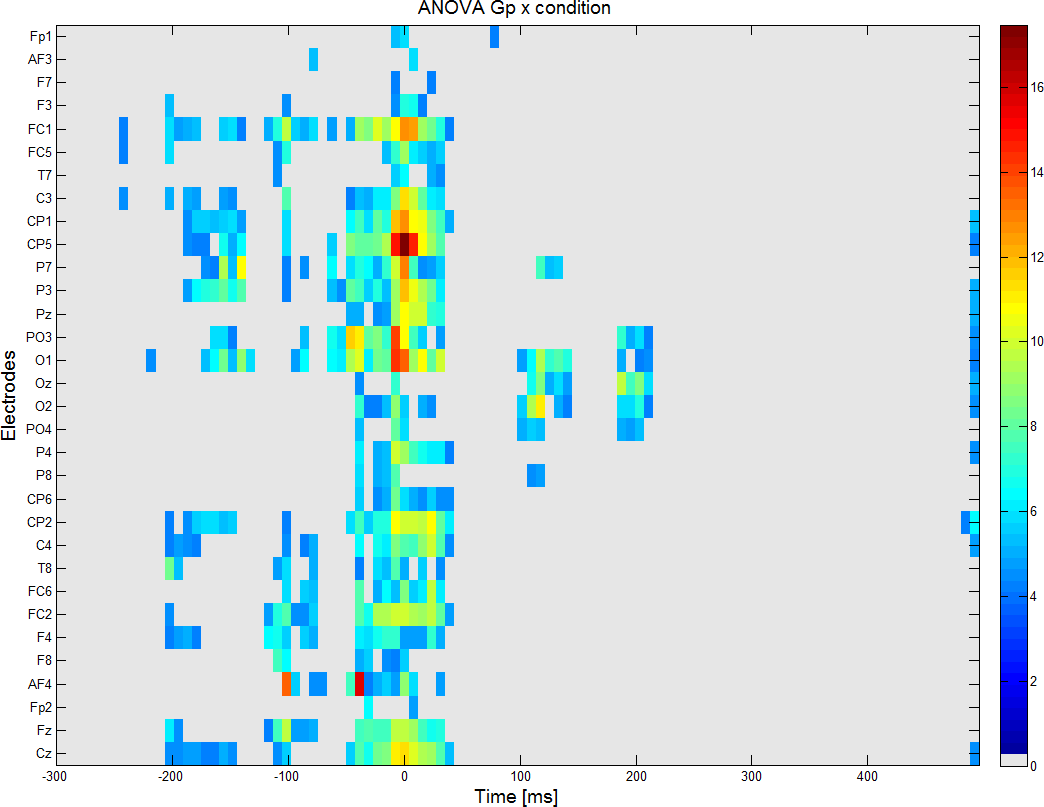


**Supplementary Figure 2.** Second level analysis in the 2 groups x 4 conditions ANOVA Values in colour for the F value explained for the TG, NG, TNG and NG conditions.


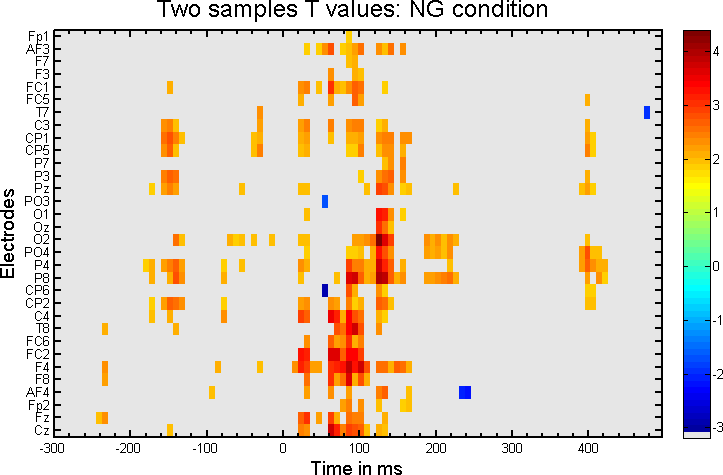


**Supplementary Figure 3.** Comparison of the Second level analysis for NG condition comparison between Control and Schizophrenic patients. Results are based on 10,000 bootstrap mean differences. Values in colour for the T value explained for the 4 conditions plus one regressor run in the first level analysis. Note that right Positive values are across several time ranges and negative values are shown from 400 to 460 ms.


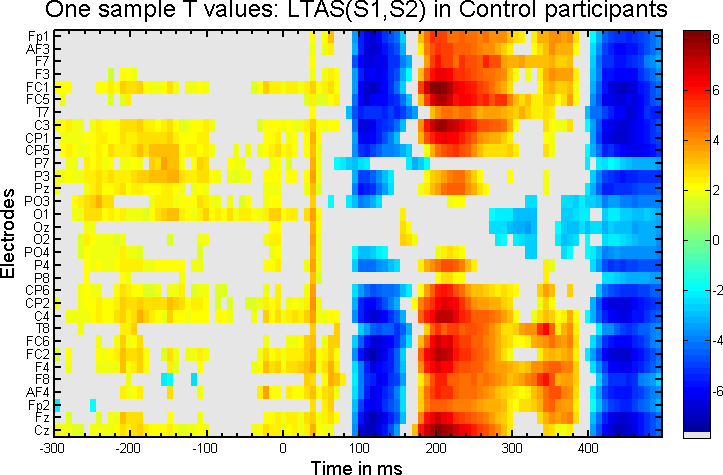


**Supplementary Figure 4.** Second level analysis for LTAS between the warning signal sound and the Target in Control participants, bootstrapped 1000 times. Values in colour for the T value explained for the 4 conditions plus one regressor run in the first level analysis. Note time range shown of negative values from 90 to 170 ms, positive values from 190 ms to 380 ms and being stronger at around 180-240 ms.
